# Application of green tea to enhance the antioxidant properties of fermented cauliflower

**DOI:** 10.1038/s41598-025-26104-y

**Published:** 2025-11-26

**Authors:** Adamska Iwona, Felisiak Katarzyna, Tokarczyk Grzegorz, Ceglarek Anna

**Affiliations:** 1 Department of Fish, Plant and Gastronomy Technology, West Pomeranian University of Technology in Szczecin, Szczecin, Poland; 2 Kamień Pomorski, Poland

**Keywords:** Fermentation, Antioxidant activity, Ascorbic acid, Acidity, Fermented vegetables, Agriculture, Biochemistry, Biotechnology, Microbiology, Plant sciences

## Abstract

Consumers and producers are constantly looking for new products with improved or modified properties. The aim of the study was to determine the effect of dried green tea leaves on the antioxidant activity and sensory properties of fermented cauliflower. The vegetable was fermented in brine with and without tea. The proximate composition, total polyphenols content, antioxidant properties, and sensory properties of fermented cauliflowers as well as brine acidity and pH were determined. Tea significantly increased their antioxidant activity (DPPH increased from 0.172 to 5.21 µM TE/g and 3.9 on days 7 and 14, respectively; TEAC—from 0.581 to 11.48 and 12.26 µM TE/g; and FRAP—from 35.1 to 225.1 and 237.5 µM TE/g, respectively), even at the lowest concentration. The highest sensory notes were obtained for samples fermented for 7 days with 3% and 4% tea additions, and for 14 days with 0.5% and 1%. The addition of tea did not negatively affect the texture of the cauliflower. The research resulted in a fermented product with increased antioxidant content. Its introduction into the human diet will increase the amount of health-promoting substances and reduce the risk of serious illnesses.

## Introduction

The increasing knowledge of consumers about the beneficial effects of fermented products on human health contributes to their popularization and growing consumption. Cabbage and cucumbers are the most commonly preserved plant materials in Eastern and Southeastern Europe. However, the search for new flavor solutions is ongoing, leading to a significant expansion of the range of fermented products to include other plant species, including cauliflower. This vegetable is valued for its mild flavor and delicate texture^1^, but some varieties are characterized by a slightly astringent or bitter taste and a sulfurous odor, which is due to a number of chemical compounds^2–4^. Cauliflower has a short shelf life after harvest at room temperature, and can be extended to just a few days in refrigerated conditions^1^. Therefore, the fermentation process of this raw material offers an interesting solution for food producers.

So far, the research conducted on cauliflower fermentation has focused on issues related to determining the effect of lactic acid fermentation on the chemical composition, including the occurrence of amino acids and vitamins B_2_, B_9_ and B_12_ in this vegetable^5^ and biogenic amines in its fermented juice^6^. In addition, aspects related to the composition of microorganisms participating in this process were studied^7^ and their probiotic potential^8^. The possibilities of using waste from cauliflower fermentation for the production of cosmetics^9^ or as animal feed^10^ have also been analyzed. However, no research has yet been conducted on the possibility of improving the antioxidant activity of fermented cauliflower as a component of the human diet by using additives with high antioxidant values during the fermentation process. This study on enriching fermented cauliflower with ingredients that increase its antioxidant activity is therefore a novelty in this field.

When preparing fermented vegetables, additional raw materials are often incorporated into the composition, and spices are used to diversify and enrich the product’s sensory characteristics^11^, rather than to improve its antioxidant activity. To date, only studies have been conducted on the use of green tea in the production of fermented vegetable kimchi. This ingredient was found to prolong the fermentation time of the product, and the final product exhibited antimutagenic effects. The experiments determined changes in the product’s pH and acidity, but did not examine their effect on the antioxidant activity of the final product^12,13^. Information on the effect of green tea and a spice mixture on the antioxidant activity of the fermented product (kimchi) can be found in patent KR20170025806A^14^. The combination of these ingredients was found to increase the content of vitamin C and total polyphenols (from 0.37 g/100 g to 0.65 g/100 g) and also increase the DPPH radical scavenging ability (from 16 to 39%).

Tea leaves contain a number of amino acids, sterols, and vitamins B2, B3, C, and E^15^. However, its most valuable compounds are flavonoids and catechins, including epicatechin, epigallocatechin, epicatechin gallate, and epigallocatechin gallate (EGCG)^16–18^. Catechins are easily soluble in water, but as the extraction period is extended, they deteriorate the sensory qualities of the extract, causing an increasing bitter and astringent taste^19^. However, a significant change in the sensory qualities of tea leaves and infusions during the fermentation process, including a loss of bitterness, has been observed. This results from changes in their chemical composition (biotransformation of compounds). Among others, a reduction in the content of tannins and catechins, which contribute to the bitter taste of tea, has been observed. They are hydrolyzed to simpler compounds (which reduces their bitterness and astringency) or transformed into new compounds, often more complex, which enriches the flavor profile with a distinctly pleasant, slightly sweet aftertaste^20–24^.

Green tea leaf extracts exhibit significant health-promoting effects: antioxidant, anti-inflammatory, and anticancer^15,25–28^, as well as antibacterial^29,30^. In this latter respect, catechins, especially EGCG, play a special role. They negatively affect, among other things, the functioning of bacterial cell membranes and the growth of these cells^29,31,32^. Strong antibacterial effects have been observed in the case of Gram-positive bacteria^29^, which also include Lactobacillus plantarum. However, previous studies show that some strains of lactic acid bacteria (including *Lactobacillus bulgaricus*, *L. plantarum* 299 V, *L. rhamnosus* LOCK900, *Lactobacillus* spp., and *Lactobacillus brevis* GTL 79) are less sensitive to the components contained in green tea compared to other bacteria, and their small doses may even promote bacterial activity^33–38^. It was found that increasing the addition of green tea during the production of fermented vegetable mixture (kimchi) may result in an extension of the fermentation time^12^, however, the action of extracts from this plant and catechins isolated from it does not completely inhibit the fermentation processes. An example is the popular fermented beverage kombucha, made from sweetened tea infusion. Traditionally, the fermentation process is carried out by a symbiotic colony of acetic acid bacteria, lactic acid bacteria, and yeast (SCOBY), but work has also been conducted to develop this product based solely on lactic acid bacteria (including *Lactobacillus* spp. and *Lactobacillus brevis* Y52)^39,40^. Similarly, fermentation of leaves and waste from green tea production for animal feed with lactic acid bacteria did not inhibit silage formation^41–44^. The varied resistance of bacterial strains to tea components results from their genetic differences, which are manifested, among others, in different activity of efflux pumps, detoxification enzymes, activity of cytoplasmic membranes, pH tolerance, and the presence and concentration of various substances in the external environment^45–47^. Therefore, in industrial processes, the selection of the appropriate strain of microorganisms and the appropriate dose of green tea is extremely important.

*Lactobacillus* bacteria change the nutritional value of fermented raw materials (enriching products with polysaccharides while reducing protein and sugar content) and the sensory quality of raw materials^48–53^, and also increase the content of certain vitamins or slow down their decomposition. This effect has been documented, for example, for vitamin C^54–56^, vitamin B2^5,57,58^, vitamin B9^5,59,60^, vitamin B12^5,61–63^, and vitamin K^64,65^. They also increase the safety of fermented products and extend their shelf life through the production of metabolites: organic acids and bacteriocins^48,49,66,67^. Some substances contained in fermented products are exogenous antioxidants. It has been proven that, when consumed regularly, they have a health-promoting effect on the human body and are an important element in the prevention of lifestyle diseases (including neurodegenerative, cancer, and cardiovascular diseases). They also slow down the aging process^48,68–70^. Currently, we are observing an increase in the incidence of lifestyle diseases, the prevalence of which is increasing in society due to stressful and unhealthy lifestyles. This leads to the ever-increasing importance of ingredients rich in exogenous antioxidants in the diet and the need to enrich traditional products with antioxidant substances. Fermented cauliflower with tea, thanks to its increased antioxidant activity and high sensory qualities, ideally meets the needs of consumers and food producers.

The aim of this article is to present the results of research on enhancing the antioxidant activity of fermented cauliflower by adding green tea leaves during the fermentation process, without losing the sensory values of this vegetable.

## Material and methods

### Materials

The research material was white cauliflower (Andromeda F1 variety; purchased in a chain store in Poland), which was then subjected to lactic acid fermentation with the addition of dried China Sencha green tea (Oxalis, Poland) in brine solution (Sól Kłodawska Kamienna Niejodowana Do Przetworów; Kopalnia Soli “Kłodawa” S.A, Poland). The starter was a culture of active *Lactobacillus plantarum* bacteria (strain LP 299v) (Serowar, Poland).

The cauliflowers weighed approximately 1.5 kg and were approximately 20 cm in diameter. They were mature (estimated age was 140–150 days after sowing), harvested two days before the experiment began. The health condition was very good: there was no discoloration, browning or damage on the inflorescences, and the leaves were fresh, green and firm.

### Experiment

The fermentation process was carried out in sterilized glass fermenters with a capacity of 500 ml. Fermenters were anaerobic (no free air access due to the tight cap being fitted and tightened). Each cap was fitted with an airlock, which served three important functions: it prevented air and microbiological contamination from entering the fermenter, vented excess CO_2_, and thus regulated the pressure within the fermenter. Sterilization of the fermenters, caps, and stoppers was always performed with steam for 3 min at 134 °C. During this process, the sterilization process was monitored physically (monitoring and recording sterilization parameters: temperature, pressure, and time) and chemically (using integrated indicator strips (Class 5). Biological monitoring of the sterilizer was performed in the laboratory once every two months and additionally after each autoclave repair.

The same *Lactobacillus plantarum* (LP 299v) starter was used in all experiments. No starter-free control was run during the studies. The initial microbiological load was 3 × 10^5^ CFU/ml. The experiment was repeated three times.

**Fig. 1:**
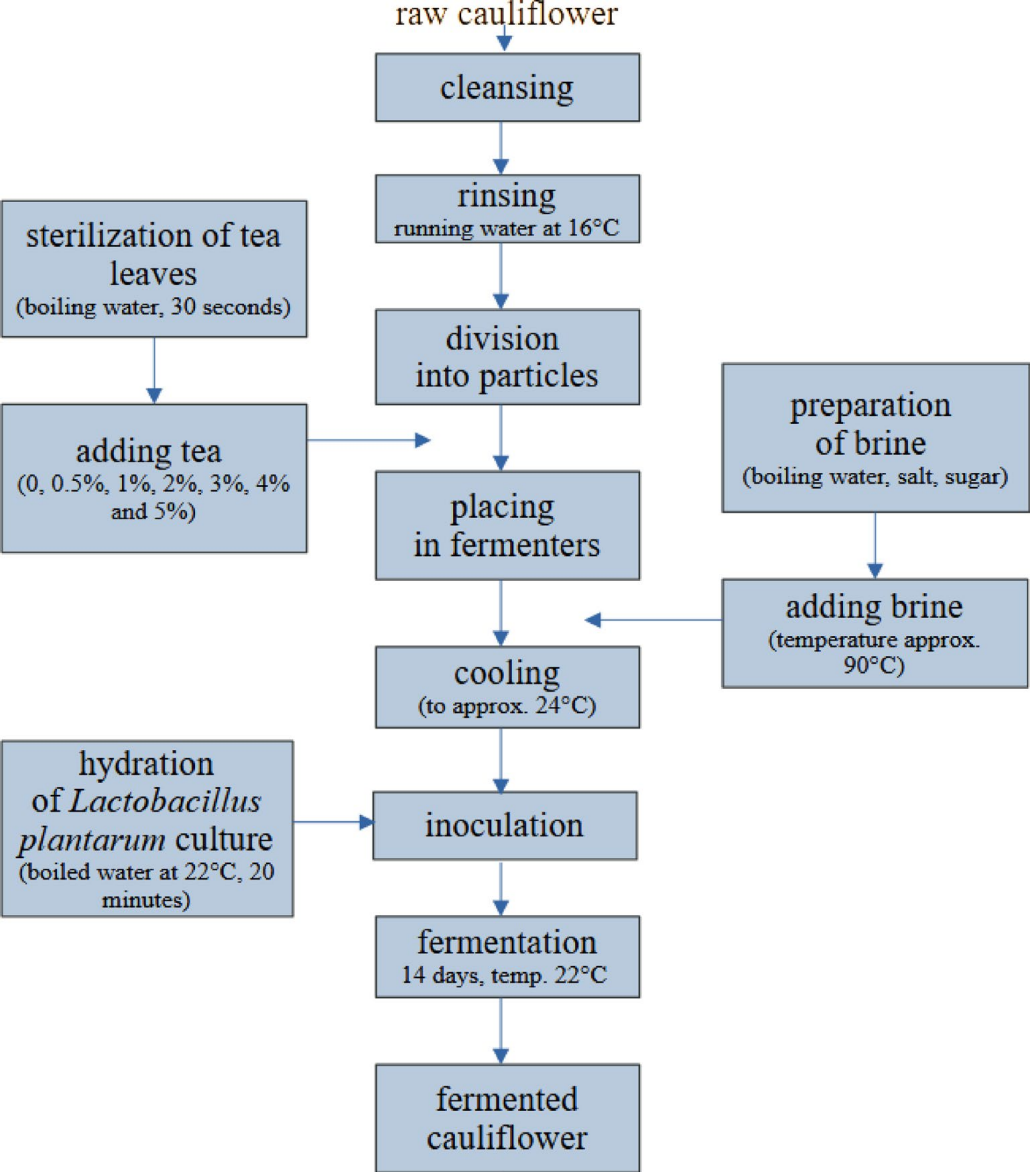
Technological scheme of experiment.

At the bottom of each container, except for the fermenters containing control samples, dried green tea leaves were placed (respectively in the amount of 0.5, 1, 2, 3, 4, and 5% of the mass of the main raw material, i.e., according to the mass of cauliflower, approximately 0.65 g, 1.3 g, 2.6 g, 3.9 g, 5.2 g, and 6.5 g, respectively), followed by cleaned, rinsed and dried cauliflower divided into small florets (approximately 130 g in each fermenter). Before being added to the fermenter, the tea leaves were sterilized (poured with boiling water for 30 s, then drained). The whole was flooded with 360 ml of hot brine prepared in the proportion of 1 l of water / 30 g of salt / 5 g of sugar. After the brine had cooled down, previously hydrated *L. plantarum* bacteria (30 ml/fermenter) were added to each fermenter and the whole was screwed with a sterilized lid. The fermentation process took place in a room with a constant temperature (22 °C) and lasted 14 days (Fig. 1). On the 7th and last day of the experiment, samples were taken from cauliflower and brine for laboratory analyses and a sensory evaluation of the cauliflower was performed.

The samples were designated according to the amount of dried green tea (GT) used in a fermenter: Control—control sample (without dried green tea), 0.5%—sample containing GT at level of 0.5% of cauliflower mass, 1%—sample containing 1% of GT, 2%—sample containing 2% GT, 3%—sample containing 3% GT, 4%—sample containing 4% GT and 5%—sample containing 5% GT. The experiment was carried out in triplicate.

### Laboratory analyses

Each physicochemical test was performed three times.

During fermentation (on day 0, 1, 4, 7, 10 and 14), the pH of brine was measured using an automatic pH meter (ChemLand PL-700 PVS, accuracy 0.02 pH). Brine was stirred before each measurement.

Samples for other physicochemical analyses were taken from fresh cauliflower (RC) on the day of experiment establishment (day 0), while samples from fermented cauliflower (Control-5%) were taken on the 7th and 14th day of fermentation. The dry matter content was determined using the thermogravimetric method, fat content using the Soxhlet method, nitrogen content using the Kjeldahl method (the conversion factor of 6.25 for protein content) and ash content using the dry ash method^71^.

The amount of carbohydrates (g/100 g ww) was calculated using formula:

$${\text{X}} = {\text{a}}{-}({\text{b}} + {\text{c}} + {\text{d}})$$

where a—dry matter content (g/100 g ww), b—fat content (g/100 g ww), c—protein content (g/100 g ww), d—ash content (g/100 g ww).

Total acidity and vitamin C content in fresh and fermented cauliflower were determined. The total acidity was determined by titration with NaOH solution (0.1 M) in the presence of an indicator (phenolphthalein), and the vitamin C content was determined by the Tillmans method^72^.

Methanolic extracts were prepared, by doubled homogenization (4000 rpm, 30 s) of cauliflower sample with methanol (5 g/50 ml w/v) with 5 min break. After 30 min of incubation at ambient temperature extracts were filtrated through medium paper filter to dark glass bottles. Total polyphenols content (TPC) was determined according to Turkmen et al.^73^, using 10% Folin–Ciocalteau reagent (5 ml) and 7.5% Na_2_CO_3_ (4 ml) added to 1 ml of diluted extract. After 2 h in the dark the absorbance was measured at 750 nm. Results were expressed as gallic acid equivalents per 1 g of wet weight. The standard curve was prepared for gallic acid concentration ranged from 0.02 to 5.0 mg/ml. Dilution of extracts was chosen to obtain absorbance not higher than 0.700 and results obtained were multiplied by dilution factor.

Total flavonoids content (TFC) was determined by combining diluted extract (2.0 ml) with 10% aluminum chloride (w/v, 0.1 ml), 1 M sodium acetate (0.1 ml), and distilled water (2.8 ml), and the absorbance at 415 nm was measured after incubation in dark for 30 min^74^. Results were expressed as quercetin equivalents per 1 g of wet weight, using standard curve prepared for 0.01 to 10 mg/ml quercetin solutions.

The antioxidant activity of cauliflower was studied in methanol extracts as free radicals DPPH scavenging ability^75^, Trolox equivalent antioxidant capacity (TEAC) against ABTS cation radicals^76^, and ferric reducing antioxidant power (FRAP)^77^. Briefly, DPPH scavenging ability (DPPH) was determined by combining diluted extract with 0.2 mM DPPH solution in methanol, and after 30 min of incubation in dark the absorbance at 517 nm was measured. The inhibition of absorbance ranged from 20 to 80% was compared with DPPH scavenging ability of Trolox (standard concentration ranged from 0.001 to 0.02 mM, corresponding to the absorbance inhibition from 3 to 80%, respectively) and expressed as µM TE/g ww. TEAC activity was determined by application of cation radical ABTS solution (7 mM ABTS activated by 2.45 mM K_2_S_2_O_8_ for 16 h), previously diluted in methanol to obtain absorbance 0.700 ± 0.020 at wavelength of 734 nm. After 30 min of incubation in dark, the absorbance was measured at the same wavelength. Results were calculated as the absorbance inhibition (ranged from 20 to 80%) and compared to the effect of Trolox (standard curve prepared for concentrations ranged from 0.02 to 2.0 mM, corresponding to the absorbance inhibition from 3 to 90%, respectively), and expressed as µM TE/g ww. In turn, FRAP was determined by combining extract with freshly prepared working solution (TPTZ in 0.04 M HCl, 0.02 M FeCl_3_ and 0.3 M acetic buffer pH 3.6 combined in ratio 1:1:10, and heated for 30 min at 37 ◦C). After 30 min of incubation the absorbance at 593 nm was measured and compared with ferric reducing ability of Trolox (standard concentration ranged from 0.002 to 0.500 mM).

### Sensory assessment

The fermented cauliflowers were subjected to a point assessment of overall sensory acceptability and assessed using the sensory profiling method. A 5-point scale was used for the assessment, where: 1—meant a very poor quality product, 2—poor, 3—moderate, 4—good, and 5—very good. In the profiling method, the assessment of previously selected descriptors was carried out according to a 10-point scale (0–9), where 0—undetectable, 1—extremely weakly detectable, 2—very weakly detectable, 3—weakly detectable, 4—slightly detectable, 5—moderately detectable, 6—detectable, 7—strongly detectable, 8—very strongly detectable, 9—extremely strongly detectable. The descriptors were: sweet, salty, sour, bitter, cauliflower, pickled cucumber, tea and other; salty, vinegary, sweet, sour, tea, pickled cucumber, bland, and other; texture crunchy, compact, rubbery, soggy, sticky, and unctuous.

The sensory panel consisted of 12 trained individuals, 6 men and 6 women, aged 22–53. Each panelist underwent a training process characterized by fixed stages: recruitment and selection of candidates (selection of candidates based on completed questionnaires and preliminary tests determining the ability to recognize sensory values), theoretical training (initial familiarization with the principles of the conducted research), methodological training and practical training (learning how to conduct sensory evaluations in practice), validation (determining the repeatability of results and consistency with the results of other panelists), qualification for the research panel, and then maintaining the high quality and suitability of the team members for research through repeated exercises (constant calibration), (according standards^78–81^). All participants gave their voluntary, informed consent to participate in the study and signed the required document. The researchers informed the participants about the purpose of the study and the anonymization of the results. Each participant had the opportunity to withdraw from the study at any stage of the study.

The sensory evaluation of the products was carried out under conditions consistent with the recommendations specified in the PN-EN ISO 8589:2010 standard^82^. The testing rooms were lit with neutral white light and isolated from noise, vibration, and undesirable odors. They were maintained at constant humidity, a controlled temperature (22 °C), and ventilation. Product samples for evaluation were prepared in separate rooms from the evaluation rooms. Samples were kept at room temperature during the evaluation. They were coded separately for each panelist with three-digit codes (each panelist received a sample with a different code to avoid the risk of misinterpreting the results of other participants). The code numbers were randomly selected and recorded before the test in a document accessible only to the person responsible for coding^83^.

Each sample weighed approximately 30 g, allowing for repeated assessment of the sensory profile in terms of the selected descriptor. They were uniform in appearance and relatively similar in shape. They were prepared simultaneously for all panelists and served in sets (each set contained one sample from each trial), with the order of the samples within each set randomized (block randomization). The samples were served on identical glass trays (odorless), without any markings, labels, or descriptions. The assessment was conducted on day 7 of fermentation and then repeated on day 14 of fermentation.

Sensory analyses were carried out in accordance with the principles specified in the PN-ISO 11035:1999, PN-EN ISO 13299:2010, PN-ISO 4121:1998, PN-EN ISO 11036:1999 standards^84–87^.

### Statistical analysis

The values presented in the tables are arithmetic means of three replicates. The results were statistically analyzed: arithmetic means were calculated for the studied variants of features, the significance of differences between them was demonstrated by the Tukey test (*P* < 0.05; two-way ANOVA analysis; the variable was the tested parameter, and the predictor of quality was fermentation time and tea dose). The relationship between the main components (antioxidant activity and bioactive compounds) was demonstrated by cluster analysis and the PCA method (Statistica 13.3, Statsoft, Tulsa, USA). Results of sensory analysis were examined using one-way ANOVA analysis (significant differences with tea dose as predictor), as well as nonparametric tests, including median, minimal and maximal values, and variance. Differences between medians for samples of the same tea concentration and different fermentation time were determined using Wilcoxon matched-pairs test (*P* < 0.05).

## Results and discussion

### Acidity and pH

During fermentation, the pH of brine decreased significantly in each experimental variant. The lowest value was observed on the last day of the experiment in sample with the addition of 4% tea. In all fermenters, the final pH remained at a similar level (Table 1), which suggests that the presence of dried green tea, even in the highest dose used, did not negatively affect the fermentation process.

**Table 1:** Changes in pH of brine during fermentation.

Day of fermentation	Green tea concentration						
	Control	0.5%	1%	2%	3%	4%	5%
0 (brine)	8.03 ± 0.01^d^	8.03 ± 0.01^d^	8.03 ± 0.01^d^	8.03 ± 0.01^d^	8.03 ± 0.01^d^	8.03 ± 0.01^d^	8.03 ± 0.01^d^
1	5.90 ± 0.40^c^	5.84 ± 0.08^c^	5.92 ± 0.01^c^	5.78 ± 0.10^c^	5.87 ± 0.09^c^	5.86 ± 0.14^c^	5.94 ± 0.63^c^
4	4.12 ± 0.37^b^	4.14 ± 0.03^b^	4.07 ± 0.04^b^	4.10 ± 0.11^b^	4.07 ± 0.06^b^	4.12 ± 0.06^b^	4.06 ± 0.09^b^
7	3.60 ± 0.32^ab^	3.44 ± 0.03^a^	3.36 ± 0.09^a^	3.26 ± 0.07^a^	3.35 ± 0.05^a^	3.24 ± 0.09^a^	3.21 ± 0.09^a^
10	3.35 ± 0.23^a^	3.19 ± 0.03^a^	3.21 ± 0.04^a^	3.12 ± 0.13^a^	3.16 ± 0.04^a^	3.11 ± 0.03^a^	3.09 ± 0.06^a^
14	3.07 ± 0.15^a^	3.08 ± 0.02^a^	3.00 ± 0.13^a^	3.04 ± 0.05^a^	3.02 ± 0.06^a^	2.99 ± 0.12^a^	3.02 ± 0.07^a^

*no statistically significant differences between values marked with the same letters in a row (n = 3).

The acidity of cauliflower and brine during the fermentation process in all tested experimental variants was higher than the acidity of raw cauliflower and fresh brine. After 7 and 14 days of fermentation, the acidity in the control sample was significantly lower than the acidity of cauliflower samples fermented with the addition of tea. In cauliflower samples fermented for 7 days, there are no significant differences in the acidity of samples with different doses of green tea. However, with the extension of fermentation time, the acidity of cauliflower increased; on day 14, it was the highest in samples with doses of 0.5–1% of tea (Table 2).

**Table 2:** Acidity of raw and fermented cauliflower and brine.

	Acidity	
	Cauliflower	Brine
Raw	0.21 ± 0.01^a^	0.00^a^

Green tea concentration	Day of fermentation			
	Cauliflower - 7	Cauliflower - 14	Brine - 7	Brine - 14
Control	0.29 ± 0.02^b^	0.31 ± 0.01^b^	0.24 ± 0.00^b^	0.43 ± 0.01^cd^
0.5%	0.50 ± 0.02^c^	0.77 ± 0.02^e^	0.45 ± 0.01^d^	0.65 ± 0.00^f^.
1%	0.46 ± 0.02^c^	0.72 ± 0.04^e^	0.44 ± 0.01^d^	0.64 ± 0.01^f^.
2%	0.49 ± 0.01^c^	0.67 ± 0.01^d^	0.44 ± 0.00b^cd^	0.64 ± 0.00^ef^
3%	0.47 ± 0.01^c^	0.64 ± 0.01^d^	0.43 ± 0.00b^cd^	0.63 ± 0.02^e^
4%	0.47 ± 0.01^c^	0.63 ± 0.02^d^	0.44 ± 0.01b^cd^	0.76 ± 0.00^h^
5%	0.47 ± 0.01^c^	0.64 ± 0.01^d^	0.42 ± 0.02^c^	0.74 ± 0.01 ^g^

*no statistically significant differences between values marked with the same letters in the columns relating to cauliflower and brine separately (regardless of the fermentation time) (n = 3).

The increase in acidity and decrease in pH of the brine during the fermentation process is caused by the formation of organic fermentation products, including lactic acid, acetic acid, and alcohol^88–91^.

### Proximate composition

The dry matter content as well as ash and carbohydrates content in fermented cauliflower were higher compared to the raw material, while the protein content was statistically significantly lower (*p* < 0.05). There was no effect of the green tea dose on the protein content in fermented cauliflowers (Table 3).

**Table 3:** The proximate composition of raw and fermented cauliflower.

	Dry matter	Protein	Ash	Fat	Carbohydrates
Content (g/100 g ww)					
Fermentation for 7 days					
Raw	6.28 ± 0.04^a^	2.63 ± 0.29^b^	0.89 ± 0.05^a^	1.38 ± 0.35^a^	1.38 ± 0.56^a^
Control	7.53 ± 0.08^c^	1.13 ± 0. 07^a^	1.98 ± 0.02^cdefg^	1.80 ± 0.17^ab^	2.62 ± 0.26^cd^
0.5%	7.12 ± 0.02^b^	1.14 ± 0.30^a^	1.77 ± 0.00^b^	1.83 ± 0.18^abc^	2.38 ± 0.33^bcd^
1%	7.29 ± 0.04^bc^	1.12 ± 0.21^a^	1.87 ± 0.10^c^	1.58 ± 0.11^ab^	2.68 ± 0.20^d^
2%	7.14 ± 0.17^bc^	1.25 ± 0.04^a^	1.89 ± 0.10^cd^	1.43 ± 0.05^a^	2.58 ± 0.28^cd^
3%	7.18 ± 0.04^bc^	1.28 ± 0.15^a^	1.98 ± 0.12^cdefg^	1.44 ± 0.03^a^	2.49 ± 0.16^bcd^
4%	7.16 ± 0.06^bc^	1.15 ± 0.12^a^	1.92 ± 0.14^cde^	1.54 ± 0.47^ab^	2.50 ± 0.39^bcd^
5%	7.09 ± 0.06^b^	1.18 ± 0.18^a^	1.94 ± 0.15^cdef^	1.44 ± 0.24^a^	2.53 ± 0.24^cd^
Fermentation for 14 days					
Control	7.27 ± 0.01^bc^	1.33 ± 0.02^a^	2.20 ± 0.01^efgh^	1.94 ± 0.06^abcd^	1.80 ± 0.08^abcd^
0.5%	7.00 ± 0.12^b^	1.23 ± 0.21^a^	2.32 ± 0.03^h^	1.99 ± 0.09^abcd^	1.46 ± 0.28^ab^
1%	6.91 ± 0.20^b^	1.16 ± 0.26^a^	2.22 ± 0.13^fgh^	1.86 ± 0.15^abc^	1.66 ± 0.31^abc^
2%	7.05 ± 0.02^b^	1.21 ± 0.17^a^	2.33 ± 0.04^h^	1.82 ± 0.04^abc^	1.70 ± 0.25^abcd^
3%	7.12 ± 0.03^b^	1.15 ± 0.17^a^	2.35 ± 0.19^h^	2.12 ± 0.25b^cd^	1.51 ± 0.24^ab^
4%	7.03 ± 0.36^b^	1.14 ± 0.17^a^	2.24 ± 0.04^gh^	2.42 ± 0.05^cd^	1.23 ± 0.16^a^
5%	7.01 ± 0.36^b^	1.10 ± 0.26^a^	2.18 ± 0.06^defgh^	2.47 ± 0.09^d^	1.27 ± 0.72^a^

*no statistically significant differences between values marked with the same letters in a column (n = 3); ww—wet weight.

According to literature data, changes occurring in raw materials and brine depend on the type of raw material and its composition, as well as on the biology of microorganisms involved in the fermentation process and on the conditions in which this process is carried out. The most frequently observed changes in the proximate composition occurring in raw materials during fermentation were a decrease in dry mass and a decrease in the amount of carbohydrates, including water-soluble sugars: glucose, fructose, and sucrose. The above-mentioned carbohydrate components were transformed by microorganisms into lactic acid, acetic acid, and propionic acids. Sometimes there were also changes in the fat and protein content and the composition of amino acids, but they were multidirectional—in some raw materials their content decreased, while in others it increased^5,90–94^.

### Bioactive compounds and antioxidant activity

In contrast to the basic components, the content of bioactive components changed significantly depending on the addition of green tea leaves.

The addition of tea leaves to the brine caused a significant increase in the content of total polyphenols already at a concentration of 0.5%. Tea leaves, unlike cauliflower, are very rich in polyphenols^16,18^, therefore increasing the amount of tea caused a further increase in the total polyphenols content in fermented cauliflowers (Table 4). After 14 days of fermentation, the TPC was lower than after 7 days. Lactic acid bacteria are relatively resistant to polyphenol content (unlike other bacteria) because they have developed mechanisms to counteract their impact, including dissociation of polyphenol-substrate complexes, inactivation of polyphenols through high-affinity binders, alteration, or degradation of phenolics^95^. These effects occur through the production of enzymes such as tannases, esterases, phenolic acid decarboxylases, and glycosidases, which modify the polyphenol profile^96^. The amount of huge polyphenols molecules like tannins decreased, however small molecular mass compounds, like catechins or gallic acid increase, therefore antioxidant activity of the fermented products increase. However, hydrolysis of glycosidic bonds occurs, resulting in increased polyphenol solubility and extractability of antioxidant compounds, therefore, polyphenol content decreases with prolonged fermentation^96^. Kim et al.^97^ demonstrated that *L. plantarum* strains resulted in significant bitterness changes and increased antioxidant, antiglycation, and anti-aggregation activities. The solubility of polyphenols increases, thus potentially increasing their solubility and recovering more antioxidant compounds^96^. Although LAB have been found to cause changes in polyphenol content during fermentation, a control sample The total content of flavonoids (TFC) in cauliflower samples fermented for 7 days was higher than in raw cauliflower, but after 14 days it decreased by approx. 50–75%, while the samples without tea or with tea added up to 2% did not differ statistically significantly. In turn, the content of ascorbic acid (AA) in samples fermented with tea added at a concentration of 1–5% did not differ statistically significantly (p < 0.05). Yang et al.^98^, who subjected fruit and vegetable juice to 14-day fermentation with *L. plantarum*, also found an increase in TFC content and antioxidant activity of DPPH, TEAC and FRAP until day 6–8, and then a decrease in these values.

**Table 4:** Bioactive compounds and antioxidant activity of fermented cauliflower.

	TPC [mg GAE/g]	TFC [µg QE/g]	AA [mg/g]	DPPH [µM TE/g]	TEAC [µM TE/g]	FRAP [µM TE/g]
Raw	0.312 ± 0.009^b^	6.01 ± 0.42^b^	0.96 ± 0.03^e^	0.487 ± 0.056^a^	3.30 ± 0.25^b^	35.3 ± 3.0^b^
Green tea concentration	Fermentation for 7 days					
Control	0.250 ± 0.016^a^	10.0 ± 0.7^c^	0.59 ± 0.03^c^	0.172 ± 0.003^a^	0.581 ± 0.24^a^	35.1 ± 2.5^b^
0.5%	0.710 ± 0.007^ef^	16.7 ± 1.6^d^	0.75 ± 0.03^d^	2.38 ± 0.09^c^	4.47 ± 0.29^b^	180.1 ± 2.0^d^
1%	0.731 ± 0.011^f^.	16.4 ± 1.4^d^	0.85 ± 0.03^d^	2.77 ± 0.31^c^	5.58 ± 0.40^c^	259.7 ± 3.1^h^
2%	0.721 ± 0.023^ef^	16.1 ± 1.2^d^	0.82 ± 0.00^d^	4.01 ± 0.31^e^	8.19 ± 0.47^e^	288.2 ± 5.1^i^
3%	0.737 ± 0.011^f.^	16.5 ± 0.3^d^	0.88 ± 0.01^de^	3.48 ± 0.73^cde^	7.57 ± 0.46^de^	292.0 ± 4.1^i^
4%	0.708 ± 0.041^ef^	19.6 ± 0.3^e^	0.89 ± 0.03^de^	4.12 ± 0.22^e^	8.55 ± 0.37^e^	310.4 ± 4.7^j^
5%	0.750 ± 0.008^f^.	19.7 ± 2.3^e^	0.89 ± 0.03^de^	5.21 ± 0.38^f^.	11.48 ± 0.27^fg^	335.1 ± 3.2^k^
	Fermentation for 14 days					
Control	0.221 ± 0.009^a^	3.35 ± 0.95^a^	0.24 ± 0.03^a^	0.156 ± 0.01^a^	0.48 ± 0.09^a^	19.8 ± 2.4^a^
0.5%	0.439 ± 0.010^c^	3.62 ± 0.57^a^	0.37 ± 0.04^b^	0.974 ± 0.10^b^	4.75 ± 0.12^bc^	130.3 ± 12.3^c^
1%	0.491 ± 0.004^c^	3.63 ± 0.48^a^	0.35 ± 0.04^b^	1.54 ± 0.14^b^	6.53 ± 0.17^cd^	191.7 ± 3.2^e^
2%	0.517 ± 0.010 ^cd^	3.72 ± 0.69^ab^	0.34 ± 0.03^b^	3.78 ± 0.21^de^	8.71 ± 0.76^e^	196.6 ± 8.9^ef^
3%	0.531 ± 0.003 ^cd^	5.46 ± 0.42^ab^	0.31 ± 0.03^ab^	3.15 ± 0.16^cd^	10.76 ± 0.52^f^.	197.8 ± 3.2^ef^
4%	0.543 ± 0.009^d^	5.11 ± 0.69^ab^	0.29 ± 0.02^ab^	3.87 ± 0.06^de^	11.93 ± 0.64^fg^	204.6 ± 3.1^f^.
5%	0.679 ± 0.010^e^	5.64 ± 0.88^ab^	0.26 ± 0.03^a^	3.96 ± 0.11^de^	12.26 ± 0.48^g^	237.5 ± 1.7^g^

*no statistically significant differences between values marked with the same letters in the column (n = 3); symbols: TPC—total phenolic compounds; TFC—total flavonoids content, AA—ascorbic acid, DPPH—DPPH radical scavenging ability, TEAC—Trolox equivalent antioxidant capacity; FRAP—ferric reducing antioxidant power.

Fermented cauliflower was characterized by several times lower antioxidant activity of DPPH and TEAC than raw cauliflower, only FRAP did not differ statistically significantly (*p* < 0.05). Water-soluble compounds which show the antioxidant activity, like ascorbic acid or some phenolic compounds have leached into the brine. On the other hand, lactic acid fermentation causes increase of low-molecular mass phenolic compounds and amino acids which shows higher antioxidant activities than macromolecules^96^. The addition of green tea leaves to the brine caused a multiple increase in antioxidant activity of fermented cauliflower, regardless of the method used (Table 4). Changes in antioxidant activity resulted from changes in the content of bioactive components (TPC, TFC, AA) and metabolites formed during fermentation. Green tea is a very rich source of polyphenols, the main ones being catechins^18^. Catechins are easily soluble in water, so they are extracted from tea leaves into the brine and can diffuse into cauliflower^19^. During fermentation, tea epigallocatechin gallate, epigallocatechin and epicatechin were hydrolysed to gallocatechin gallate and gallocatechin^22,99^. Many authors have confirmed that during fermentation their amount decreased^22,23,100^. Enzymes responsible for the hydrolysis of polyphenols lead to the formation of simpler phenolic compounds and their aglycones or conjugated glycoside forms, which proves the bioavailability and functionality of the obtained products^101,102^.

### Sensory evaluation and acceptability

**Fig. 2:**
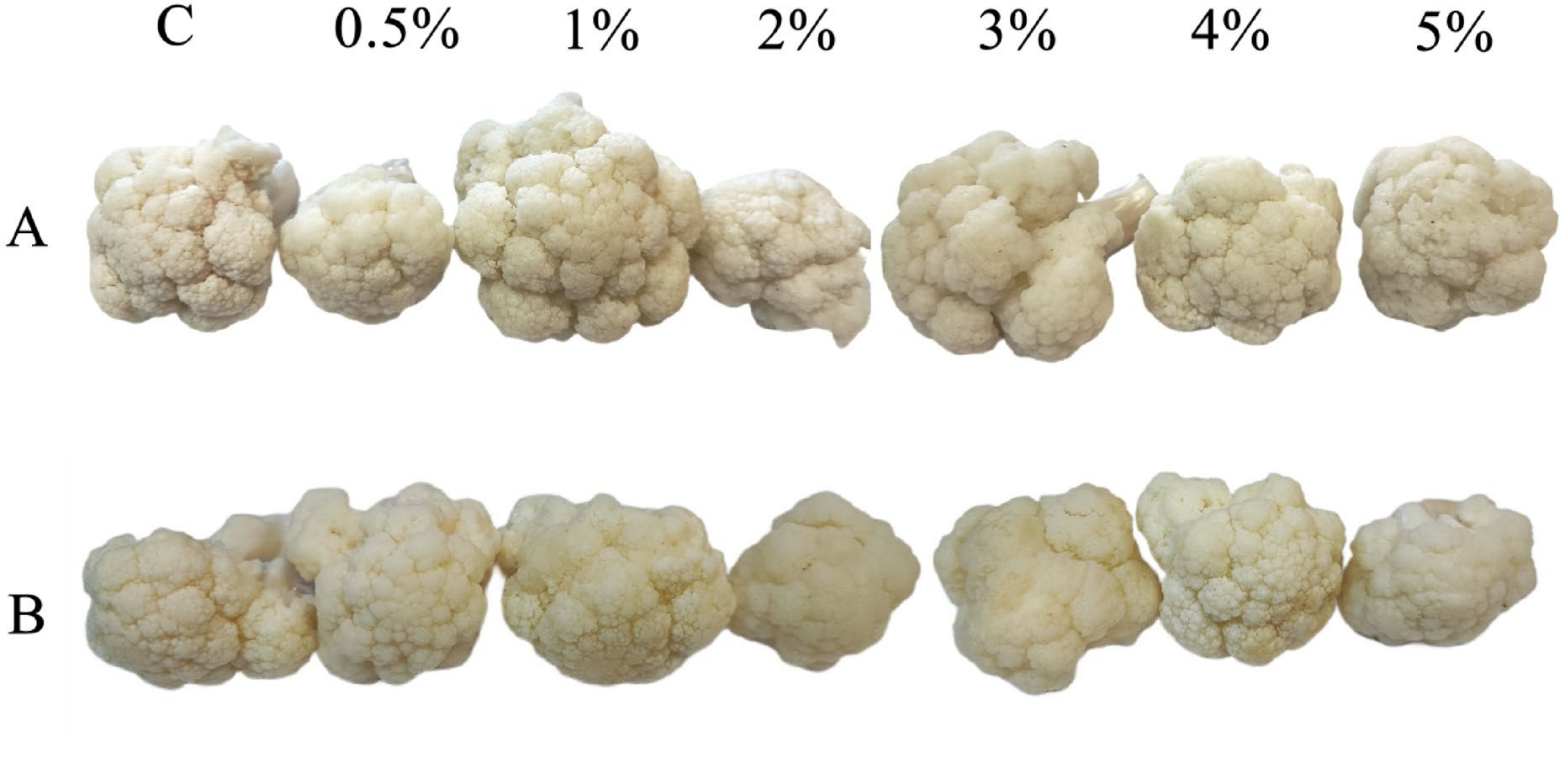
Fermented cauliflowers on 7th (**A**) and 14th (**B**) day of fermentation. Symbols: C—control sample (without dried green tea), 0.5%—sample containing GT at level of 0.5% of cauliflower mass, 1%—sample containing 1% of GT, 2%—sample containing 2% GT, 3%—sample containing 3% GT, 4%—sample containing 4% GT and 5%—sample containing 5% GT.

In this study, fermenting cauliflower for 14 days with higher doses of dried tea (4% and 5%) resulted in a slight color change to light yellow (Fig. 2). This was likely due to the effect of the tea leaf extract, which lightens and clarifies during fermentation^100^.

**Fig. 3:**
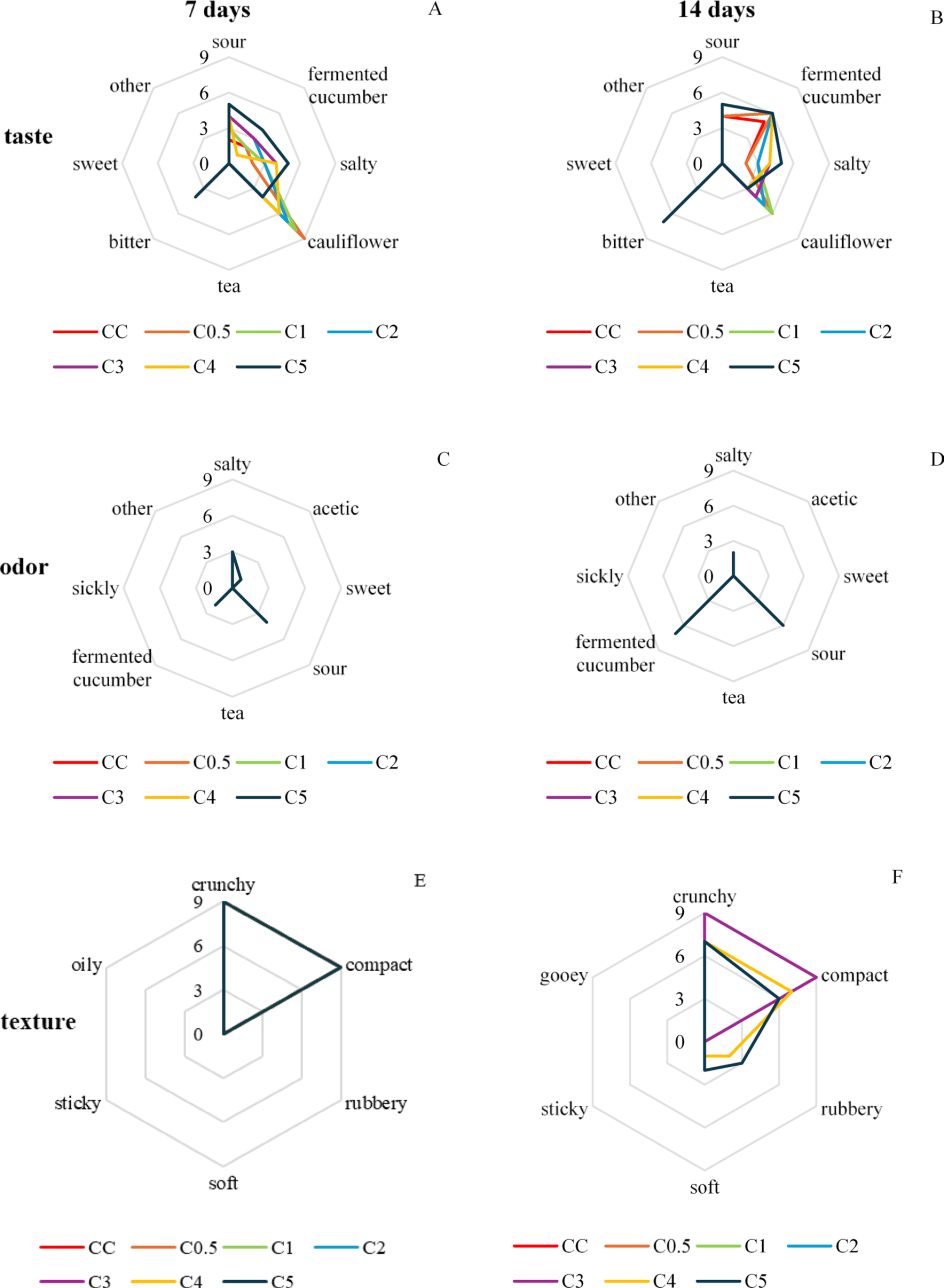
Profiles of taste, odor, and texture of fermented cauliflower. (**A**) Taste, fermentation for 7 days, (**B**) Taste, fermentation for 14, (**C**) Odor, fermentation for 7 days, (**D**) Odor, fermentation for 14 days, (**E**) Texture, fermentation for 7 days, (**F**) Texture, fermentation for 14 days. Symbols: CC—control sample (without dried green tea), C0.5—sample containing GT at level of 0.5% of cauliflower mass, C1—sample containing 1% of GT, C2—sample containing 2% GT, C3—sample containing 3% GT, C4—sample containing 4% GT and C5—sample containing 5% GT.

On the 14th day of fermentation, the best sensory scores were obtained for cauliflower samples fermented with 0.5% and 1% dried tea (Table 5). The cauliflower had a salty-sour taste, typical of cauliflower and pickled cucumbers, and an aroma typical of pickled cucumbers (it was the most noticeable aroma), additionally sour and slightly salty (Fig. 3A–D).

**Table 5:** Overall sensory acceptability of fermented cauliflower determined by point method.

Green tea concentration	Mean	Median	Min	Max	Variance
Fermentation for 7 days					
Control	3.5 ± 0.5^a^*	3.0^A^**	3	4	0.265
0.5%	3.5 ± 0.7^a^	3.0^A^	3	5	0.455
1%	3.7 ± 0.7^a^	4.0^A^	3	5	0.424
2%	3.9 ± 0.7^ab^	4.0^A^	3	5	0.447
3%	4.9 ± 0.3^c^	5.0^A^	4	5	0.083
4%	4.8 ± 0.4^c^	5.0^A^	4	5	0.152
5%	4.6 ± 0.5^bc^	5.0^A^	4	5	0.265
Fermentation for 14 days					
Control	4.0 ± 0.6^c^	4.0^B^	3	5	0.364
0.5%	5.0 ± 0.1^d^	5.0^B^	4.5	5	0.021
1%	4.7 ± 0.5^d^	5.0^B^	4	5	0.203
2%	4.0 ± 0.6^c^	4.0^A^	3	5	0.364
3%	3.7 ± 0.5^c^	4.0^B^	3	4	0.242
4%	1.8 ± 0.7^b^	2.0^B^	1	3	0.430
5%	1.0 ± 0.1^a^	1.0^B^	1	1.5	0.021

*no statistically significant differences between means of the samples with different tea concentration after the same time of fermentation marked with the same lower-case in a column (n = 12).

**no statistically significant differences between medians of the samples with the same tea concentration after 7 and 14 days of fermentation marked with the same capital letters in a column (n = 12).

Green tea water extracts prepared with long brewing times are characterized by a very intense, bitter taste. In this study, the bitter taste was clearly noticeable only in samples fermented for 14 days with more than 1% tea (Fig. 3A,B). Products fermented with green tea leaves may be characterized by a bitter taste, but usually this impression is much weaker than in the case of a long-brewed tea infusion. The reason for this can be chemical changes occurring both in green tea and in the infusion itself. During fermentation, the content of ingredients that affect the taste of tea is reduced. This causes the taste to become stronger, but less bitter, sour–sweet^20–23,100,103^ with a noticeable umami aftertaste^104^. During fermentation, the content of tannins^105^, catechins^22,23,100^ and sucrose^103^ decreases. In addition, polyphenols are oxidized^22,100^, proteins are hydrolyzed, which are converted into amino acids^62,70^. In infusions, the content of simple sugars increases^100^ and new phenolic acids and catechin derivatives are produced^23^. The acids produced, including lactic acid^103^, cause a decrease in pH^22,100,103^.

According to the literature, tea fermentation also changes its odor to a milder, floral-fruity one, which is caused by the ketones, aldehydes and esters formed^21,24,100,106,107^. However, in this research, odor was most clearly noticeable with the notes of the fermented main raw material (cauliflower), similar to the odor of a pickled cucumber, while tea or fruity-floral notes were completely imperceptible. The odor components of fermented brassicas (including cauliflower) are mainly products resulting from the degradation of glucosinolates^108,109^, and the aroma profile of fermented cabbage consists of as many as 61 volatile compounds, including, among others, esters, terpenes, acids, alcohols, sulfur compounds, carbonyl compounds, and nitriles^110–113^. Fermented vegetable products also contain diactetyl, a component responsible for the characteristic buttery flavor and odor of the products^102^.

Changes in the texture of fermented cauliflower in this study were observed only on day 14 of fermentation in the 4% and 5% tea samples (Fig. 3E,F). Vegetables subjected to the fermentation process (including yellow beetroot and kimchi mixture) changed their texture: a decrease in their hardness, elasticity, and chewiness was observed in comparison with raw materials. The softening of plant tissues resulting from the influence of salt was the reason. This occurred as a result of cell swelling, disruption of membranes (plasmolysis) and cell walls in plant tissues by water accumulating in them, and then leakage of cell contents into the brine^114,115^.

### PCA analysis

**Fig. 4:**
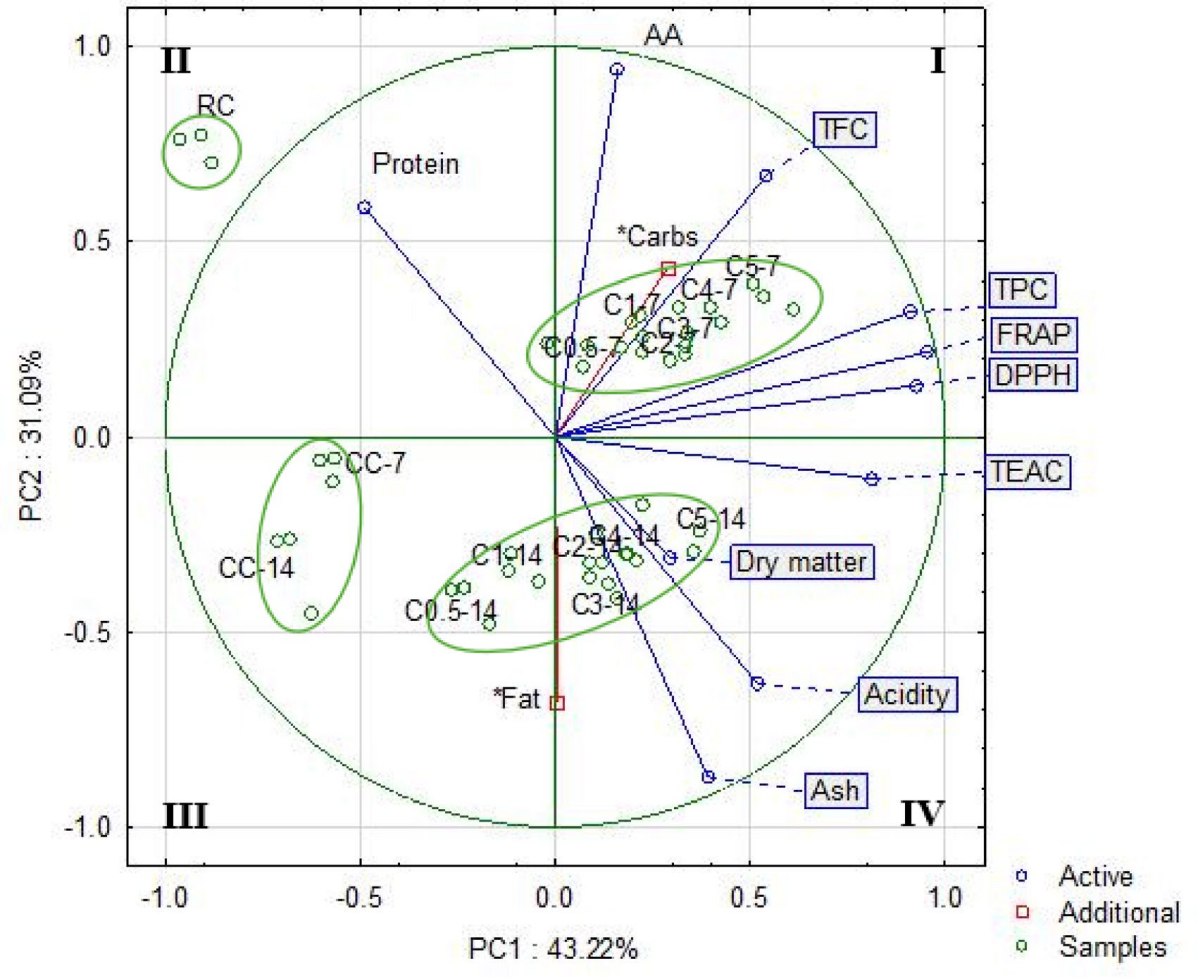
PCA biplot of the first two principal components for bioactive compounds, antioxidant activities, and proximate components of fermented cauliflowers. TPC, total phenolic compounds; TFC, total flavonoids content; AA, ascorbic acid; TEAC, Trolox equivalent antioxidant capacity; FRAP, ferric reducing antioxidant power; DPPH, DPPH radical scavenging ability; RC, fresh cauliflower; n = 3; Symbols: CC—control sample (without dried green tea), C0.5—sample containing GT at level of 0.5% of cauliflower mass, C1—sample containing 1% of GT, C2—sample containing 2% GT, C3—sample containing 3% GT, C4—sample containing 4% GT and C5—sample containing 5% GT.

Principal components analysis showed that the two initial components explained 74.31% of the variance, while PC1 explained 43.22%. The antioxidant activity determined by all methods correlated significantly with total phenolic compounds, and to a lesser extent with flavonoid content (Fig. 4). A weak correlation was found between them and ascorbic acid content, as well as the content of basic components, especially fat content.

The distribution of samples in the quadrants of a two-factor case coordinate chart showed samples mutual correlations. Samples found in quadrant I are strongly correlated with high levels of bioactive compounds and antioxidant activities. There are cauliflowers with tea addition fermented for 7 days. Fresh cauliflower constituted a separate cluster, furthest from the others, located in the second quadrant, indicating a negative correlation with antioxidants content and properties. Cauliflower samples fermented without the addition of tea for 7 and 14 days formed the cluster in the third quadrant, while cauliflower samples fermented with the addition of green tea for 14 days formed another cluster in III and IV quadrants, indicating an increase in the strength of correlation with antioxidant properties with increasing tea concentration in the samples.

## Summary

Fermenting cauliflower in brine with the addition of tea leaves enriches this traditional product with ingredients that increase its bioactivity. This is an alternative to traditional fermented products: it supplements the diet with minerals and antioxidants. It enhances the positive effects of fermented vegetables on human health and may contribute to improved dietary antioxidant intake.

Green tea increases polyphenols and antioxidant activity. The addition of tea did not significantly change the nutritional value of fermented cauliflower, its color, or crispness. The optimal concentration of 0.5–1% is to maintain sensory quality. The most recommended dose of tea leaves is 0.5–1% of cauliflower weight and fermentation lasting 14 days. The research results may have potential application in the development of functional fermented products.

One limitation of this study was the small number of panelists. Research conducted on a larger sample, taking into account differences by gender, age, education, and place of residence, most accurately reflects the perception of the product among general consumers. A second limitation was that only chemical and sensory analysis was conducted (no biological testing). Biological tests would demonstrate the product’s usefulness in the diet of healthy people and people with various diseases. Therefore, further research primarily involves microbiological testing of the product, which will determine whether it affects the effectiveness of reference strains of microorganisms (e.g., *Bacillus cereus, B. subtilis, Enterococcus faecalis, Escherichia coli, Listeria monocytogenes* or *Salmonella enteritidis*). Our next planned study is a bioavailability test and cellular cytotoxicity testing on selected cancer cell lines, such as the LoVo line of human colon cancer. This would allow us to assess whether the product can support therapeutic processes. Conducting a comprehensive consumer study will also be crucial to determine whether the product, with our chosen green tea dose and fermentation time, will appeal to a wider consumer audience.

## Data Availability

The datasets used and/or analysed during the current study available from the corresponding author on reasonable request.

## References

[1] Jarmoluk, A. Food technology. *T.1. Raw materials, products, functional food* (in Polish) (Educational Publishing House WSiP, 2016).

[2] Engel, E., Baty, C., Le Corre, D., Souchon, I. & Martin, N. Flavor-active compounds potentially implicated in cooked cauliflower acceptance. *J. Agric. Food Chem.* **50**, 6459–6467. 10.1021/jf025579u (2002). 12381134 10.1021/jf025579u

[3] Bell, L., Oloyede, O. O., Lignou, S., Wagstaff, C. & Methven, L. Taste and flavor perceptions of glucosinolates, isothiocyanates, and related compounds. *Mol. Nutr. Food Res.* **62**, 1700990. 10.1002/mnfr.201700990 (2018). 10.1002/mnfr.201700990 29578640

[4] Wieczorek, M. N. et al. The relation between phytochemical composition and sensory traits of selected *Brassica* vegetables. *LWT* **156**, 113028. 10.1016/j.lwt.2021.113028 (2022).

[5] Thompson, H. O., Önning, G., Holmgren, K., Strandler, H. S. & Hultberg, M. Fermentation of cauliflower and white beans with *Lactobacillus plantarum*—Impact on levels of riboflavin, folate, vitamin B12, and amino acid composition. *Plant Foods Hum. Nutr.* **75**, 236–242. 10.1007/s11130-020-00806-2 (2020). 32144644 10.1007/s11130-020-00806-2 PMC7266841

[6] Jastrzębska, A. et al. Determination of selected biogenic amines in fermented vegetables juices. *Food Control* **154**, 109980. 10.1016/j.foodcont.2023.109980 (2023).

[7] Paramithiotis, S., Hondrodimou, O. L. & Drosinos, E. H. Development of the microbial community during spontaneous cauliflower fermentation. *Food Res. Int.* **43**, 1098–1103. 10.1016/j.foodres.2010.01.023 (2010).

[8] Rachwał, K., Gustaw, K. & Sadok, I. Enhancing food sustainability through probiotics isolated from fermented cauliflower. *Sustainability* **16**, 8340. 10.3390/su16198340 (2024).

[9] Herman, A., Matulewicz, O., Korzeniowska, E. & Herman, A. P. Determination of post-fermentation waste from fermented vegetables as potential substitutes for preservatives in o/w emulsion. *Int. J. Mol. Sci.* **25**, 5510. 10.3390/ijms25105510 (2024). 38791548 10.3390/ijms25105510 PMC11122242

[10] Xu, H., Wu, H., Zhou, R., Yu, F. & Zang, R. The effects of fermented cauliflower residue feed on the diarrhea rate, intestinal morphology, immune indicators, and intestinal flora of weaned piglets. *Fermentation* **10**, 465. 10.3390/fermentation10090465 (2024).

[11] Wojdyła, T. & Wichrowska, D. The influence of additives used and storage methods on the quality of sauerkraut. *Inż. Ap. Chem.* **53**, 424–426 (2014).

[12] Choi, W.-Y. & Park, K.-Y. Increased preservative and antimutagenic activities of kimchi with addition of green tea leaves. *J. Food Sci. Nat.* **5**, 189–193 (2000).

[13] Patent KR100384309B1. Functional Kimchi added green tea and process for preparation thereof (Korea, 2003).

[14] Patent KR20170025806A. Kimchi Composition Comprising Vitamin C and Its Antioxidative Activity and Whitening Effect (Korea, 2017).

[15] Musial, C., Kuban-Jankowska, A. & Gorska-Ponikowska, M. Beneficial properties of green tea catechins. *Int. J. Mol. Sci.* **21**, 1744. 10.3390/ijms21051744 (2020). 32143309 10.3390/ijms21051744 PMC7084675

[16] Cichoń, Z., Miśniakiewicz, M. & Szkudlarek, E. Properties of green tea (in Polish). *Zesz. Nauk.* **73**, 59–90 (2007).

[17] Kodama, D. H., Goncalves, A. E. S. S., Lajolo, F. M. & Genovese, M. I. Flavonoids, total phenolics and antioxidant capacity: Comparison between commercial green tea preparations. *Ciênc. Tecnol. Aliment. Camp.* **30**, 1077–1082. 10.1590/S0101-20612010000400037 (2010).

[18] Luo, Q. et al. Green extraction of antioxidant polyphenols from green tea (*Camellia sinensis*). *Antioxidants* **9**, 785. 10.3390/antiox9090785 (2020). 32854245 10.3390/antiox9090785 PMC7555212

[19] Narukawa, M. et al. Evaluation of the bitterness of green tea catechins by a cell-based assay with the human bitter taste receptor hTAS2R39. *Biochem. Biophys. Res. Commun.* **405**, 620–625. 10.1016/j.bbrc.2011.01.079 (2011). 21272567 10.1016/j.bbrc.2011.01.079

[20] Han, T. & Aye, K. N. The legend of laphet: A Myanmar fermented tea leaf. *J. Ethn. Foods* **2**, 173–178. 10.1016/j.jef.2015.11.003 (2015).

[21] Cao, L. T. et al. A comparative analysis for the volatile compounds of various Chinese dark teas using combinatory metabolomics and fungal solid-state fermentation. *J. Food Drug Anal.* **26**, 112–123. 10.1016/j.jfda.2016.11.020 (2018). 29389546 10.1016/j.jfda.2016.11.020 PMC9332658

[22] Zhao, Z.-J. et al. Flavour chemical dynamics during fermentation of kombucha tea. *EJFA* **30**, 732–741. 10.9755/ejfa.2018.v30.i9.1794 (2018).

[23] Cheng, L. et al. Distinct changes of metabolic profile and sensory quality during Qingzhuan tea processing revealed by LC-MS-Based metabolomics. *J. Agri. Food Chem.* **68**, 4955–4965. 10.1021/acs.jafc.0c00581 (2020). 10.1021/acs.jafc.0c00581 32286813

[24] Wang, R., Sun, J. C., Lassabliere, B., Yu, B. & Liu, S. Q. UPLC-Q-TOF-MS based metabolomics and chemometric analyses for green tea fermented with *Saccharomyces boulardii* CNCM I-745 and *Lactiplantibacillus plantarum* 299V. *Curr. Res. Food Sci.* **5**, 471–478. 10.1016/j.crfs.2022.02.012 (2022). 35252880 10.1016/j.crfs.2022.02.012 PMC8892000

[25] Bogdański, P. et al. Green tea extract reduces blood pressure, inflammatory biomarkers, and oxidative stress and improves parameters associated with insulin resistance in obese, hypertensive patients. *Nutr. Res.* **32**, 421–427. 10.1016/j.nutres.2012.05.007 (2012). 22749178 10.1016/j.nutres.2012.05.007

[26] Indarti, K., Apriani, E. F., Wibowo, A. E. & Simanjuntak, P. Antioxidant activity of ethanolic extract and various fractions from green tea (*Camellia**sinensis* L.) leaves. *Pharmacog. J.* **11**, 771–776. 10.5530/pj.2019.11.122 (2019).

[27] Xu, X. Y. et al. Effects and mechanisms of tea for the prevention and management of cancers: An updated review. *Crit. Rev. Food Sci. Nutr.* **60**, 1693–1705. 10.1080/10408398.2019.1588223 (2020). 30869995 10.1080/10408398.2019.1588223

[28] Hirose, K. et al. Preparing and characterizing of xyloglucan films containing tea extract for oral mucositis. *ACS Omega* **10**, 390–399. 10.1021/acsomega.4c06410 (2025). 39829602 10.1021/acsomega.4c06410 PMC11740623

[29] Liu, S., Zhang, Q., Li, H., Qiu, Z. & Yu, Y. Comparative assessment of the antibacterial efficacies and mechanisms of different tea extracts. *Foods* **11**, 620. 10.3390/foods11040620 (2022). 35206096 10.3390/foods11040620 PMC8870964

[30] Alkufeidy, R. M., Altuwijri, L. A., Aldosari, N. S., Alsakabi, N. & Dawoud, T. M. Antimicrobial and synergistic properties of green tea catechins against microbial pathogens. *J. King Saud Univ. Sci.* **36**, 103277. 10.1016/j.jksus.2024.103277 (2024).

[31] Parvez, Md. A. K. et al. Antibacterial activities of green tea crude extracts and synergistic effects of epigallocatechingallate (EGCG) with gentamicin against MDR pathogens. *Heliyon* **5**, e02126. 10.1016/j.heliyon.2019.e02126 (2019). 31372566 10.1016/j.heliyon.2019.e02126 PMC6658803

[32] Kováć, J., Slobodníková, L., Nebus, B. & Kurin, E. Antibacterial activity of green tea and peppermint extracts against *Enterococcus**faecalis* and the potential of EGCG in oral health. *Eur. Pharm. J.* **2025**, 1–7. 10.2478/afpuc-2025-0002 (2025).

[33] McCue, P. P. & Shetty, K. Phenolic antioxidant mobilization during yogurt production from soymilk using Kefir cultures. *Process Biochem.* **40**, 1791–1797 (2005).

[34] Lee, H. C., Jenner, A. M., Low, C. S. & Lee, Y. K. Effect of tea phenolics and their aromatic fecal bacterial metabolites on intestinal microbiota. *Res. Microbiol.* **157**, 876–884 (2006). 16962743 10.1016/j.resmic.2006.07.004

[35] Jaziri, I., Ben Slama, M., Mhadhbi, H., Urdaci, M. C. & Hamdi, M. Effect of green and black teas (*Camellia**sinensis* L.) on the characteristic microflora of yogurt during fermentation and refrigerated storage. *Food Chem.* **112**, 614–620 (2009).

[36] Neffe-Skocińska, K., Jaworska, D., Kołożyn-Krajewska, D., Dolatowski, Z. & Jachacz-Jówko, L. The effect of LAB as probiotic starter culture and green tea extract addition on dry fermented pork loins quality. *Biomed. Res. Int.* **19**, 452757. 10.1155/2015/452757 (2015). 10.1155/2015/452757 PMC4417591 25961018

[37] Jin, Y. H. et al. Lactic acid fermented green tea with *Levilactobacillus* brevis capable of producing γ-aminobutyric acid. *Fermentation* **7**, 110. 10.3390/fermentation7030110 (2021).

[38] Wang, R., Sun, J., Lassabliere, B., Yu, B. & Liu, S. Green tea fermentation with *Saccharomyces**boulardii* CNCM I-745 and *Lactiplantibacillus**plantarum* 299V. *LWT* **157**, 113081. 10.1016/j.lwt.2022.113081 (2024). 10.1016/j.crfs.2022.02.012 PMC8892000 35252880

[39] Nguyen, N. K., Dong, N. T., Nguyen, H. & Le, P. H. Lactic acid bacteria: Promising supplements for enhancing the biological activities of Kombucha. *Springer Plus* **4**, 91. 10.1186/s40064-015-0872-3 (2015). 25763303 10.1186/s40064-015-0872-3 PMC4348356

[40] Xia, X.-X., Wang, B. & Fang, F. Enhancement of Kombucha fermentation by adding lactic acid bacteria. *Food Ferment. Ind.* **44**, 185–192. 10.13995/j.cnki.11-1802/ts.017688 (2018).

[41] Zhuang, X. et al. Fermentation quality of herbal tea residue and its application in fattening cattle under heat stress. *BMC Vet. Res.* **17**, 348. 10.1186/s12917-021-03061-y (2021). 34772402 10.1186/s12917-021-03061-y PMC8588620

[42] Chen, X., Zhou, X., Li, S., Zhang, H. & Liu., Z.,. Effects of tea residues-fermented feed on production performance, egg quality, antioxidant capacity, caecal microbiota, and ammonia emissions of laying hens. *Front. Vet. Sci.* **10**, 1195074. 10.3389/fvets.2023.1195074 (2023). 37426079 10.3389/fvets.2023.1195074 PMC10325031

[43] Hong, G.-H., Lee, S.-Y., Yoo, J.-I., Chung, J. H. & Park, K.-Y. Catechin with lactic acid bacteria starters enhances the antiobesity effect of kimchi. *J. Med. Food* **26**, 560–569. 10.1089/jmf.2023.K.0067 (2023). 37405755 10.1089/jmf.2023.K.0067

[44] Hong, G.-H., Lee, S.-Y. & Park, K.-Y. Antiobesity effect and metabolite analysis of catechin functional kimchi. *J. Ethn. Foods* **11**, 32. 10.1186/s42779-024-00248-0 (2024).

[45] Hayashi, T., Ueda, S., Suruta, H. T., Kuwahara, H. & Osawa, R. Complexing of green tea Catechins with food constituents and degradation of the complexes by *Lactobacillus plantarum*. *BMFH* **31**, 27–36. 10.12938/bmfh.31.27 (2012). 24936346 10.12938/bmfh.31.27 PMC4034289

[46] Tarrah, A. et al. Probiotic potential and biofilm inhibitory activity of *Lactobacillus casei* group strains isolated from infant feces. *J. Funct. Foods* **54**, 489–497. 10.1016/j.jff.2019.02.004 (2019).

[47] Tumbarski, Y. et al. Characterization and selection of *Lactobacillus* strains with potential probiotic applications. *Appl. Sci.* **15**, 2902. 10.3390/app15062902 (2025).

[48] Anumudu, C. K., Miri, T. & Onyeaka, H. Multifunctional applications of lactic acid bacteria: Enhancing safety, quality, and nutritional value in foods and fermented beverages. *Foods* **13**, 3714. 10.3390/foods13233714 (2024). 39682785 10.3390/foods13233714 PMC11640447

[49] Bangar, S. P., Suri, S., Trif, M. & Ozogul, F. Organic acids production from lactic acid bacteria: A preservation approach. *Food Biosci.* **46**, 101615. 10.1016/j.fbio.2022.101615 (2022).

[50] Ravyts, F., Vuyst, L. D. & Leroy, F. Bacterial diversity and functionalities in food fermentations. *Eng. Life Sci.* **12**, 356–367. 10.1002/elsc.201100119 (2012).

[51] Zielinski, H., Surma, M. & Zielinska, D. The naturally fermented sour pickled cucumbers. In *Fermented Foods in Health and Disease Prevention* (eds Frias, J. et al.) 503–516 (Academic Press, Cambridge, 2017).

[52] Daba, G. M., Elnahas, M. O. & Elkhateeb, W. A. Contributions of exopolysaccharides from lactic acid bacteria as biotechnological tools in food, pharmaceutical, and medical applications. *Int. J. Biol. Macromol.* **173**, 79–89. 10.1016/j.ijbiomac.2021.01.110 (2021). 33482209 10.1016/j.ijbiomac.2021.01.110

[53] Korcz, E. & Varga, L. Exopolysaccharides from lactic acid bacteria: Techno-functional application in the food industry. *Trends Food Sci. Technol.* **110**, 375–384. 10.1016/j.tifs.2021.02.014 (2021).

[54] Kuria, M. W., Matofari, J. W. & Nduko, J. M. Physicochemical, antioxidant, and sensory properties of functional mango (*Mangifera**indica* L.) leather fermented by lactic acid bacteria. *J. Agric. Food Res.* **6**, 100206. 10.1016/j.jafr.2021.100206 (2021).

[55] Quan, Q., Liu, W., Guo, J., Ye, M. & Zhang, J. Effect of six lactic acid bacteria strains on physicochemical characteristics, antioxidant activities and sensory properties of fermented orange juices. *Foods* **11**, 1920. 10.3390/foods11131920 (2022). 35804736 10.3390/foods11131920 PMC9265423

[56] Xu, H. et al. Change of phytochemicals and bioactive substances in *Lactobacillus* fermented *Citrus* juice during the fermentation process. *LWT* **180**, 114715. 10.1016/j.lwt.2023.114715 (2023).

[57] Jayashree, S., Jayaraman, K. & Kalaichelvan, G. Isolation, screening and characterization of riboflavin producting lactic acid bacteria from Katpadi Vellore district. *Recent Res. Sci. Technol.* **2**, 83–88 (2010).

[58] Capozzi, V. et al. Biotechnological production of vitamin B2-enriched bread and pasta. *J. Agric. Food Chem.* **59**, 8013–8020. 10.1021/jf201519h (2011). 21678896 10.1021/jf201519h

[59] Santos, F., Wegkamp, A., de Vos, W. M., Smid, E. J. & Hugenholtz, J. High-level folate production in fermented foods by the B12 producer *Lactobacillus reuteri* JCM1112. *Appl. Environ. Microbiol.* **74**, 3291–3294. 10.1128/AEM.02719-07 (2008). 18344331 10.1128/AEM.02719-07 PMC2394963

[60] LeBlanc, J. G., Taranto, M. P., Molina, V. & Sesma, F. B-group vitamins production by probiotic lactic acid bacteria in *Biotechnology of Lactic Acid Bacteria: Novel Applications* (eds. Mozzi, F., Raya, R., Vignolo G.) 211–232 (Wiley-Blackwell 2010).

[61] Torres, A. C. et al. Cobalamin production by *Lactobacillus coryniformis*: Biochemical identification of the synthetized corrinoid and genomic analysis of the biosynthetic cluster. *BMC Microbiol.* **16**, 240. 10.1186/s12866-016-0854-9 (2016). 27737643 10.1186/s12866-016-0854-9 PMC5064896

[62] Li, P., Gu, Q., Yang, L., Yu, Y. & Wang, Y. Characterization of extracellular vitamin B12 producing *Lactobacillus plantarum* strains and assessment of the probiotic potentials. *Food Chem.* **234**, 494–501. 10.1016/j.foodchem.2017.05.037 (2017). 28551266 10.1016/j.foodchem.2017.05.037

[63] Singh, B. & Sharma, S. Vitamin B12 production by *Lactobacillus* species isolated from milk products. *J. Res. Appl. Sci. Biotechnol.* **1**, 48–59. 10.55544/jrasb.1.2.6 (2022).

[64] Morishita, T., Tamura, N., Makino, T. & Kudo, S. Production of menaquinones by lactic acid bacteria. *J. Dairy Sci.* **82**, 1897–1903 (1999). 10509247 10.3168/jds.S0022-0302(99)75424-X

[65] Boe, C. A. & Holo, H. Engineering *Lactococcus lactis* for Increased Vitamin K2 Production. *Front Bioeng. Biotechnol.* **8**, 191. 10.3389/fbioe.2020.00191 (2020). 32258010 10.3389/fbioe.2020.00191 PMC7093718

[66] Behera, S. S., Ray, R. C. & Zdolec, N. *Lactobacillus plantarum* with functional properties: an approach to increase safety and shelf-life of fermented foods. *BioMed. Res. Int.* **2018**, 9361614. 10.1155/2018/9361614 (2018). 29998137 10.1155/2018/9361614 PMC5994577

[67] Tang, H., Huang, W. & Yao, Y.-F. The metabolites of lactic acid bacteria: classification, biosynthesis and modulation of gut microbiota. *Microbial Cell* **10**, 49–62. 10.15698/mic2023.03.792 (2023). 36908281 10.15698/mic2023.03.792 PMC9993431

[68] Knez, E., Kadac-Czapska, K. & Grembecka, M. Fermented vegetables and legumes vs lifestyle diseases: microbiota and more. *Life* **13**, 1044. 10.3390/life13041044 (2023). 37109573 10.3390/life13041044 PMC10141223

[69] Shah, A. M., Tarfeen, N., Mohamed, H. & Song, Y. Fermented foods: Their health-promoting components and potential effects on gut microbiota. *Fermentation* **9**, 118. 10.3390/fermentation9020118 (2023).

[70] Todorovic, S. et al. Health benefits and risks of fermented foods—the PIMENTO initiative. *Front. Nutr.* **11**, 1458536. 10.3389/fnut.2024.1458536 (2024). 39309142 10.3389/fnut.2024.1458536 PMC11414650

[71] AOAC. *Official Method of Analysis, 18th ed*. (Association of Officiating Analytical Chemists, 2015).

[72] Krełowska-Kułas, M. *Analyses of Food Products Quality* (in polish) (PWE, 1993).

[73] Turkmen, N., Sari, F. & Velioglu, Y. S. The effect of cooking methods on total phenolics and antioxidant activity of selected green vegetables. *Food Chem.* **93**, 713–718. 10.1016/j.foodchem.2004.12.038 (2005).

[74] Shraim, A. M., Ahmed, T. A., Rahman, M. M. & Hijji, Y. M. Determination of total flavonoid content by aluminum chloride assay: A critical evaluation. *LWT* **150**, 111932. 10.1016/j.lwt.2021.111932 (2021).

[75] Brand-Williams, W., Cuvelier, M. E. & Berset, C. Use of a free radical method to evaluate antioxidant activity. *LWT* **28**, 25–30. 10.1016/S0023-6438(95)80008-5 (1995).

[76] Re, R. et al. Antioxidant activity applying an improved ABTS radical cation decolorization assay. *Free Radic. Biol. Med.* **26**, 1231–1237. 10.1016/S0891-5849(98)00315-3 (1999). 10381194 10.1016/s0891-5849(98)00315-3

[77] Benzie, I. F. F. & Strain, J. J. The ferric reducing ability of plasma (FRAP) as a measure of ‘Antioxidant Power’: The FRAP Assay. *Anal. Biochem.* **239**, 70–76. 10.1006/abio.1996.0292 (1996). 8660627 10.1006/abio.1996.0292

[78] PN-EN ISO 8586:2014-03 Sensory analysis. General guidelines for the selection, training, and monitoring of selected assessors and sensory evaluation experts. Polish Committee for Standardization.

[79] PN-ISO 5496:1997 Sensory analysis. Methodology. Introduction and training of assessors in the detection and recognition of odors. Polish Committee for Standardization.

[80] PN-ISO 3972:2016-07 Sensory analysis. Methodology. Methods for testing taste sensitivity. Polish Committee for Standardization

[81] PN-EN ISO 11132:2017-08 Sensory analysis. Methodology. Guidelines for monitoring the performance of a quantitative sensory system. Polish Committee for Standardization.

[82] PN-EN ISO 8589:2010 Sensory analysis. General guidelines for the design of sensory analysis laboratories. Polish Committee for Standardization.

[83] PN-ISO 5497:1998 Sensory analysis. Methodology. Guidelines for the preparation of samples for which direct sensory analysis is not possible. Polish Committee for Standardization.

[84] PN-ISO 11035:1999 Sensory analysis. Identification and selection of descriptors for determining the sensory profile using multivariate methods. Polish Committee for Standardization.

[85] PN-EN ISO 13299:2010 Sensory analysis—Methodology—General guidelines for determining sensory profiles. Polish Committee for Standardization.

[86] PN-ISO 4121:1998 Sensory analysis—Methodology—Evaluation of food products using scaling methods. Polish Committee for Standardization.

[87] PN-EN ISO 11036:1999 Sensory analysis. Methodology. Texture profiling. Polish Committee for Standardization

[88] Migut, D., Gorzelany, J. & Wołowiec, A. Evaluation of selected chemical properties of fresh and pickled field cucumbers. *Inż. Przetw. Spoż. Pol. J. Food Eng.* **3**, 33–39 (2018).

[89] Kao, C.-C. & Lin, J.-Y. Culture condition optimization of naturally lacto-fermented cucumbers based on changes in detrimental and functional ingredients. *Food Chem. X* **19**, 100839. 10.1016/j.fochx.2023.100839 (2023). 37780341 10.1016/j.fochx.2023.100839 PMC10534157

[90] Kiczorowski, P., Kiczorowska, B., Samolińska, W., Szmigielski, M. & Winiarska-Mieczan, A. Effect of fermentation of chosen vegetables on the nutrient, mineral, and biocomponent profile in human and animal nutrition. *Sci. Rep.* **12**, 13422. 10.1038/s41598-022-17782-z (2022). 35927577 10.1038/s41598-022-17782-z PMC9352655

[91] Zhou, X. et al. Dynamic changes in physic-chemical properties and bacterial community during natural fermentation of tomatoes. *Food Sci. Technol. Caminas* **42**, e63520. 10.1590/fst.63520 (2022).

[92] Ghosh, D. Studies on the changes of biochemical, microbiological and sensory parameters of sauerkraut and fermented mix vegetables. *Food Res.* **5**, 78–83. 10.26656/fr.2017.5(1).193 (2021).

[93] Singhal, P., Satya, S. & Naik, S. N. Fermented bamboo shoots: a complete nutritional, anti-nutritional and antioxidant profile of the sustainable and functional food to food security. *Food Chem.* **3**, 100041. 10.1016/j.fochms.2021.100041 (2021). 10.1016/j.fochms.2021.100041 PMC8991591 35415653

[94] Ye, J.-H., Huang, L.-Y., Terefe, N. S. & Augustin, M. A. Fermentation-based biotransformation of glucosinolates, phenolics and sugars in retorted broccoli puree by lactic acid bacteria. *Food Chem.* **286**, 616–623. 10.1016/j.foodchem.2019.02.030 (2019). 30827654 10.1016/j.foodchem.2019.02.030

[95] Dissanayake, I. H. et al. Lactic acid bacterial fermentation as a biotransformation strategy to enhance the bioavailability of phenolic antioxidants in fruits and vegetables: A comprehensive review. *Food Res. Int.* **209**, 116283. 10.1016/j.foodres.2025.116283 (2025). 40253191 10.1016/j.foodres.2025.116283

[96] Yang, F. et al. Effects of fermentation on bioactivity and the composition of polyphenols contained in polyphenol-rich foods: A review. *Foods* **12**(17), 3315 (2023). 37685247 10.3390/foods12173315 PMC10486714

[97] Kim, G. Y. et al. Synergistic antioxidant and anti-inflammatory activities of kale juice fermented with Limosilactobacills reuteri EFEL6901 or Limosilactobacills fermentum EFEL6800. *Antioxidants* **12**(10), 1850 (2023). 37891929 10.3390/antiox12101850 PMC10604225

[98] Yang, X. et al. Antioxidant properties of a vegetable–fruit beverage fermented with two *Lactobacillus plantarum* strains. *Food Sci. Biotech.* **27**, 1719–1726. 10.1007/s10068-018-0411-4 (2018). 10.1007/s10068-018-0411-4 PMC6233392 30483436

[99] Park, S.B., Han, B.K., Oh, H.J., Lee, SJ, Cha, S.K., Park, Y.S., & Choi, H.J. Bioconversion of green tea extract using lactic acid bacteria. *Food Eng. Prog.* **16,** 26–32 (2012).

[100] Hu, T., Shi, S. & Ma, Q. Modulation effects of microorganisms on tea in fermentation. *Front. Nutr.* **9**, 931790. 10.3389/fnut.2022.931790 (2022). 35983492 10.3389/fnut.2022.931790 PMC9378870

[101] Lee, N.-K. & Paik, H.-D. Bioconversion using lactic acid bacteria: ginsenosides, GABA, and phenolic compounds. *J. Microbiol. Biotechnol.* **27**, 869–877. 10.4014/jmb.1612.12005 (2017). 28297748 10.4014/jmb.1612.12005

[102] RuizRodríguez, L. G. et al. Fruits and fruit by-products as sources of bioactive compounds. Benefits and trends of lactic acid fermentation in the development of novel fruit-based functional beverages. *Food Res. Int.* **140**, 109854. 10.1016/j.foodres.2020.109854 (2021). 33648172 10.1016/j.foodres.2020.109854

[103] Cvetković, D. et al. Survival of wild strains of *Lactobacilli* during Kombucha fermentation and their contribution to functional characteristics of beverage. *Pol. J. Food Nutr. Sci.* **69**, 407–415. 10.31883/pjfns/112276 (2019).

[104] Nishioka, H., Ohno, T., Iwahashi, H. & Horie, M. Diversity of Lactic acid bacteria involved in the fermentation of Awa-bancha. *Microbes Environ.* **36**, ME21029. 10.1264/jsme2.ME21029 (2021). 34840198 10.1264/jsme2.ME21029 PMC8674441

[105] Nout, M. J. R. & Ngoddy, P. O. Technological aspects of preparing affordable fermented complementary foods. *Food Control* **8**, 279–287 (1997).

[106] Wang, R., Sun, J. C., Lassabliere, B., Yu, B. & Liu, S. Q. Fermentation characteristics of four non-Saccharomyces yeasts in green tea slurry. *Food Microbiol.* **92**, 03609. 10.1016/j.fm.2020.103609 (2020). 10.1016/j.fm.2020.103609 32950144

[107] Wang, R., Sun, J. C., Lassabliere, B., Yu, B. & Liu, S. Q. 13-Glucosidase activity of *Cyberlindnera* (*Williopsis*) *saturnus* var. mrakii NCYC 2251 and its fermentation effect on green tea aroma compounds. *LWT* **151**, 112184. 10.1016/j.lwt.2021.112184 (2021).

[108] Martinez-Villaluenga, C. et al. Influence of fermentation conditions on glucosinolates, ascorbigen, and ascorbic acid content in white cabbage (*Brassica**oleracea* var. *capitata* cv. *taler*) cultivated in different seasons. *J. Food Sci.* **74**, C62–C67. 10.1111/j.1750-3841.2008.01017.x (2009). 19200088 10.1111/j.1750-3841.2008.01017.x

[109] Ciska, E., Honke, J. & Drabińska, N. Changes in glucosinolates and their breakdown products during the fermentation of cabbage and prolonged storage of sauerkraut: focus on sauerkraut juice. *Food Chem.* **365**, 130498. 10.1016/j.foodchem.2021.130498 (2021). 34243119 10.1016/j.foodchem.2021.130498

[110] Yang, X. et al. Microbial community dynamics and metabolome changes during spontaneous fermentation of northeast sauerkraut from different households. *Front. Microbiol.* **11**, 1878. 10.3389/fmicb.2020.01878 (2020). 32849461 10.3389/fmicb.2020.01878 PMC7419431

[111] Yang, X. et al. Comparison of northeast sauerkraut fermentation between single lactic acid bacteria strains and traditional fermentation. *Food Res. Int.* **137**, 109553. 10.1016/j.foodres.2020.109553 (2020). 33233175 10.1016/j.foodres.2020.109553

[112] Satora, P., Skotniczny, M., Strnad, S. & Piechowicz, W. Chemical composition and sensory quality of sauerkraut produced from different cabbage varieties. *LWT* **136**, 110325. 10.1016/j.lwt.2020.110325 (2021).

[113] Major, N. et al. Bioactive properties, volatile compounds, and sensory profile of sauerkraut are dependent on cultivar choice and storage conditions. *Foods* **11**, 1218. 10.3390/foods11091218 (2022). 35563941 10.3390/foods11091218 PMC9101451

[114] Janiszewska-Turak, E., Kołakowska, W., Pobiega, K. & Gramza-Michałowska, A. Influence of drying type of selected fermented vegetables pomace on the natural colorants and concentration of lactic acid bacteria. *Appl. Sci.* **11**, 7864. 10.3390/app11177864 (2021).

[115] Yang, H. I. et al. Influence of salt concentration on Kimchi cabbage (*Brassica**rapa* L. ssp. *pekinensis*) mass transfer kinetics and textural and microstructural properties during osmotic dehydration. *J. Food Sci.* **88**, 1610–1622. 10.1111/1750-3841.16514 (2023). 36922723 10.1111/1750-3841.16514

